# Sexual functioning after total versus subtotal laparoscopic hysterectomy—long term follow up results after 7 years

**DOI:** 10.1007/s00404-026-08479-z

**Published:** 2026-06-07

**Authors:** Tobias Brodkorb, Ahmed Elnahrawy, Saskia Spaich, Marc Sütterlin, Sebastian Berlit, Benjamin Tuschy, Lukas Goerdt

**Affiliations:** 1 Department of Gynecology and Obstetrics, Medical Faculty Mannheim, University Medical Center Mannheim, Heidelberg University, Mannheim, Germany; 2 Department of Gynecology and Obstetrics, RKH Hospital Ludwigsburg, Ludwigsburg, Germany

**Keywords:** Laparoscopic hysterectomy, Sexual functioning, Cervix, Supracervical hysterectomy

## Abstract

**Purpose:**

The aim of this study was to evaluate sexual function in the long term after total laparoscopic hysterectomy (TLH) versus laparoscopic supracervical hysterectomy (LASH) in patients with benign indications.

**Methods:**

As part of a longitudinal follow-up, 74 patients who had originally participated in a prospective study on sexual function after TLH or LASH were surveyed again (TLH: n = 45; LASH: n = 29). The median follow-up period was 92 months (TLH) and 98 months (LASH). Sexual function was assessed using the validated Female Sexual Function Index (FSFI). Statistical analyses were performed using a 2 sample t test, Chi^2^ test and Fisher’s exact test.

**Results:**

Over the long term, there was no significant difference in the FSFI total score between TLH and LASH (22.51 vs. 22.25, p-value: 0.261). Within the TLH group, sexual function remained stable compared to baseline data (p = 0.20). In contrast, the LASH group showed a significant deterioration in the total score (p = 0.028), particularly in the lubrication (p = 0.007) over time.

**Conclusion:**

Both surgical methods ensure comparable sexual function. While TLH shows stable results over the years, LASH shows a significant decrease in some areas of sexual function, particularly with regard to lubrication. However, these differences do not result in a significantly worse overall outcome compared to TLH.

## What does this study adds to the clinical work

**Table:** 

There are no significant differences in sexual function as measured by the total score of the FSFI questionnaire between total and subtotal laparoscopic hysterectomy in the long time follow-up	

## Introduction

Hysterectomy for benign indications remains one of the most common major surgical procedures in gynecology worldwide [1–6]. Laparoscopic hysterectomy, both total laparoscopic hysterectomy (TLH) and subtotal laparoscopic hysterectomy (LASH), continue to be the most commonly performed types of hysterectomy for benign indications in Germany and have been the standard procedures for uterine removal for many years [2, 3, 7]. Benign indications for hysterectomy are manifold and range from abnormal uterine bleeding to uterine fibroids, endometriosis, and adenomyosis to genital prolapse, chronic pelvic pain, and others [8–12]. One of the most common fears among patients is postoperative sexual dysfunction [13, 14]. Possible causes for varying degrees of sexual function after surgery could be related to the preservation or removal of the cervix. With LASH, the cervix is preserved, leaving both the pelvic floor and the vagina unchanged in their anatomical structure. In contrast, with TLH, the cervix is removed, which can lead to a shortening of the vagina and an overall change in sensation [15–17]. It is also conceivable that TLH may affect more nerves in the area of the cervix and parametrium, which could result in reduced lubrication, for example [18–20]. However, it is also possible that pain will persist due to the cervix remaining in place, and sexual function may also be impaired by the possibility of continued cervical bleeding, even though there are studies that have shown the opposite [21]. Psychological aspects must also be taken into account. Some women associate the removal of the uterus with or without the cervix with a loss of femininity or sexuality, which can of course affect sexual function [22–26]. Other women, experience significant relief after the operation due to the elimination of preexisting symptoms such as pain or heavy vaginal bleeding [21]. In our previous work, we have shown that there is no significant difference in postoperative sexual function between patients who have undergone TLH or LASH due to benign conditions, and further studies have confirmed this [27–31]. In our current work, we want to look at the long-term results and determine whether, even years after the operation, there is still no significant difference in sexual function between the two surgical methods.

## Methods

This study represents the long-term follow-up to our initial work: “Sexual functioning after total versus subtotal laparoscopic hysterectomy” [28]. The study was approved by the Local Ethics Committee II of the Mannheim Medical Faculty of Heidelberg University (2012–602 N-MA). A total of 120 patients were included in the original study, for whom a total of 92 complete data sets were available, 46 of which were for TLH and 46 for LASH. For our long-term follow-up, a total of 92 patients were contacted again and 74 complete data sets are available from this group, 45 of which are for TLH and 29 for LASH. The median follow-up period was 98 months in the LASH group and 92 months in the TLH group. The choice of surgical method was left to the patients themselves in the spirit of shared decision making. The exact process for this is described in detail in our previous work. Sexual function was also assessed in this study using the standardized Female Sexual Functioning Index (FSFI). This index uses six categories to assess sexual function. The index assigns a value to each subcategory, which are then added together to form a total score. The total score can range from 2 to 36. The higher the score, the better the sexual function. A value below 26.55 is considered an indicator of sexual dysfunction. Demographic data were also collected, including age, body mass index, and whether the participant separated from their steady partner during the follow-up period.

### Statistical analysis

Quantitative variables are presented by mean value and standard deviation. Furthermore, 95% confidence intervals and effect sizes according to Cohen have been calculated. For qualitative factors, absolute frequencies and percentages are given.

In order to compare two mean values (i.e. to compare LASH and TLH) a 2 sample t test has been performed. For each surgical method, a comparison of sexual function at baseline and in the long-term follow-up was performed using a t-test for paired samples. For the comparison of percentages between two groups a Chi^2^ test has been used. If the conditions of this test were not fulfilled, Fisher’s exact test has been performed instead.

A test result with a p-value < 0.05 was considered statistically significant. The statistical analyses were performed using SPSS® (version 29; SPSS Inc., USA).

## Results

Of the total of 92 patients for whom complete baseline questionnaires were available and who were contacted again as part of this follow-up, 74 complete questionnaires were available for evaluation (TLH: 45, LASH: 29). The reasons for dropout were missing or incomplete questionnaires.

Table 1 provides a detailed overview of the demographic data, including a re-listing of the indications and complications initially recorded in relation to the primary surgery.

**Table 1:** Initial study demographic and operative data

Variable	LASH Mean ± SD	TLH Mean ± SD	p-value
Initial study demographic and operative data (LASH *n* = 46, TLH *n* = 46)			
Age (years)	46.7 ± 5.2	46.8 ± 7.3	0.935
BMI (kg/m^2^)	24.5 ± 4.1	26.7 ± 5.9	0.043
Parity	± 0.96	1.5 ± 1.0	0.029
Married	27 (59%)	36 (78%)	0.043
Stable relationship	42 (91%)	45 (98%)	0.361
Indication for surgery			0.104
Fibroid	35 (76%)	25 (54%)	0.074
Endometriosis	3 (6.5%)	3 (6.5%)	
Prophylactic	1 (2%)	1 (2%)	
Severe cervical intraepithelial neoplasia/endometrial hyperplasia	0 (0%)	4 (8.7%)	
Bleeding disorder	4 (8.7%)	11 (23.9%)	
Others	3 (6.5%)	2 (4.3%)	
Duration of surgery (h)	1.6 ± 0.68	1.9 ± 0.98	0.109
Blood loss (mL)	65 ± 77	60 ± 132	0.820
Haemoglobin drop (g/dL)	1.1 ± 0.84	1.2 ± 0.66	0.493
Uterine weight (g)	274 ± 225	182 ± 103	0.007
Number of abdominal incisions	2.1 ± 0.3	2.3 ± 0.4	0.640
Duration of hospital stay	1.8 ± 1.1	2.4 ± 0.7	0.02
Long term follow up demographic data (LASH n = 29, TLH n = 45)			
Variable	LASH Mean ± SD	TLH Mean ± SD	p-value
Age (years)	54.17 ± 3.74	55.89 ± 6.50	0.201
BMI (kg/m^2^)	27.04 ± 3.04	26.52 ± 3.16	0.487

This table contains some data that has already been published in an earlier study[28]

### Total score

The results of the evaluation of the FSFI follow-up questionnaires show no statistically significant difference in the total score between TLH and LASH patients (TLH: 22.5 ± 9.1, LASH: 22.3 ± 9.6, p = 0.910) for the long-term follow-up. A comparison of the baseline values with the follow-up data within the TLH group shows no statistically significant change (baseline: 24.7 ± 9.1, follow-up: 22.5 ± 9.7, change: 2.18 ± 11.27, p = 0.200). However, when looking at the LASH group (baseline vs. follow-up), there is a statistically significant deterioration in the total score (baseline: 27.6 ± 8.9, follow-up: 22.3 ± 9.6, change: 5.36 ± 12.50, p = 0.028). But when comparing the changes from baseline to follow-up between the groups no statistically significant difference between TLH and LASH can be detected (p = 0.261). The effect sizes according to Cohen (defined as the ratio “mean change / standard deviation”) are 0.194 (TLH) and 0.428 (LASH) indicating a small and a medium effect, respectively.

### Items of the questionnaire

Furthermore, for the LASH group a significant deterioration in lubrication (baseline: 4.96 ± 1.94, follow-up: 3.62 ± 1.87, p = 0.007) could be demonstrated.

Looking at the effect sizes it becomes obvious that the changes “baseline to Follow Up” are rather small in each group and for each item. Only for the parameter “lubrication” a medium effect (0.535) can be observed (Tables 2 and 3). The results presented in Table 4 show that there are no significant differences between the two groups regarding changes from baseline to follow up.

**Table 2:** TLH (Changes from Baseline to Follow Up)

	Long-term follow up results (TLH: n = 45)					
Variable	TLH Baseline Mean ± SD	TLH Follow Up Mean ± SD	Change Mean ± SD	95% confidence interval for change	Effect size	BL vs. FU p-value
Desire	3.50 ± 1.28	3.22 ± 1.35	0.29 ± 1.56	[-0.18, + 0.75]	0.184	0.223
Arousal	4.21 ± 1.54	3.66 ± 1.90	0.55 ± 1.99	[-0.05, + 1.14]	0.277	0.070
Lubrication	4.32 ± 2.08	3.90 ± 1.95	0.41 ± 2.43	[-0.32, + 1.14]	0.170	0.259
Orgasm	4.15 ± 1.89	3.59 ± 2.00	0.56 ± 2.48	[-0.18, + 1.35]	0.226	0.136
Satisfaction	4.51 ± 1.93	4.04 ± 1.82	0.46 ± 2.39	[-0.26, + 1.18]	0.194	0.201
Pain	3.93 ± 2.23	4.05 ± 2.29	-0.12 ± 2.79	[-0.96, + 0.72]	-0.044	0.766
Total score	24.70 ± 9.15	22.51 ± 9.68	2.18 ± 11.27	[-1.20,5.57]	0.194	0.200

**Table 3:** LASH (Changes from Baseline to Follow Up)

Variable	LASH Baseline Mean ± SD	LASH Follow Up Mean ± SD	Change Mean ± SD	95% confidence interval for change	Effect Sizes	BL vs. FU p-value
Desire	3.72 ± 0.98	3.19 ± 1.27	0.53 ± 1.45	[-0.02, + 1.08]	0.366	0.059
Arousal	4.66 ± 1.50	3.84 ± 1.88	0.82 ± 2.36	[-0.08, + 1.71]	0.347	0.073
Lubrication	4.96 ± 1.94	3.62 ± 1.87	1.34 ± 2.50	[+ 0.39, + 2.29]	0.535	0.007
Orgasm	4.65 ± 1.87	3.90 ± 1.84	0.74 ± 2.33	[-0.14, + 1.63]	0.319	0.097
Satisfaction	4.63 ± 1.83	3.69 ± 1.84	0.95 ± 2.54	[-0.02, + 1.91]	0.374	0.054
Pain	4.90 ± 1.96	4.01 ± 2.19	0.88 ± 1.06	[-0.28, + 2.05]	0.289	0.131
Total score	27.61 ± 8.93	22.25 ± 9.59	5.36 ± 12.50	[+ 0.60, + 10.11]	0.429	0.028

**Table 4:** TLH vs. LASH: Comparison of Change

Variable	Difference of changes LASH vs. TLH	95% confidence interval for difference	Changes LASH vs TLH p value	Effect Size LASH vs TLH
Desire	0.24 ± 1.52	[-0.48, + 0.96]	0.501	0.161
Arousal	0.27 ± 2.13	[-0.74, + 1.28]	0.599	0.126
Lubrication	0.92 ± 2.46	[+ 0.24, + 2.09]	0.118	0.377
Orgasm	0.18 ± 2.43	[+ 0.97, + 1.33]	0.753	0.075
Satisfaction	0.49 ± 2.45	[-0.68, + 1.65]	0.407	0.199
Pain	1.01 ± 2.89	[-0.40, + 2.42]	0.148	0.348
Total score	3.17 ± 11.77	[-2.41, + 8.76]	0.261	0.270

In summary it should be noted that only in the LASH group two significant effects have been observed: lubrication and total score, each from baseline to follow up (p = 0.007 and 0.028, respectively).

Furthermore, we performed an analysis of covariance with the outcome “total score change from baseline to follow up” in order to adjust for the baseline value. This analysis revealed a highly significant impact of the baseline value (p < 0.0001) and no impact for the group (p = 0.692). Very similar results have been obtained when performing analyses of covariance for the parameters of the questionnaire (baseline value: each p < 0.001, group: each p > 0.35).

Detailed lists of all results collected are provided in Tables 2.

Patients were asked two additional questions as part of this survey. First, they were asked whether they saw any additional factors that had influenced their sexual function. A total of 48 (64.9%) patients stated that their sexual function had been affected, 24 (32.4%) due to a lack of a sexual partner, 13 (17.6%) due to loss of sexual desire, and 11 (14.9%) due to dyspareunia. A total of 26 (35.1%) of the patients stated that their sexual function was not affected by additional factors. The patients were also asked whether they would have the original procedure performed again. All 74 (100%) patients, i.e., all patients from both the TLH and LASH groups, stated that they would have the procedure performed again.

## Discussion

The aim of this study was to investigate whether there is a difference in the sexual function of patients after TLH or LASH in long-term follow-up. There are various hypotheses as to why either surgical method could have a better outcome in terms of sexual function. The cervix is at the center of these considerations. On the one hand, the cervix is believed to have a potential effect on sexual arousal and genital sensation; on the other hand, the cervix is associated with dyspareunia and deep pain during sexual intercourse [32–36]. Potentially, the presence of a cervix offers the possibility of both promoting sexual function and, in individual cases, inhibiting it [33, 37]. Further considerations concern, for example, a shortened vagina after TLH and the resulting dyspareunia and psychological effects [38, 39]. The psychological aspects focus primarily on the woman's self-image. For many women, the preservation of the female reproductive organs plays an important role in their self-image and their sense of being a complete woman. It is therefore conceivable that the removal of the female reproductive organs could also have a negative effect on sexual function through a reduced self-image or self-esteem. The extent to which these effects could differ in terms of self-image and thus sexual function in cases of partial removal of the uterus, with at least the cervix remaining in place as part of LASH, is difficult to assess due to the highly individual nature of perception in this regard. What we (do) know is that quality of life and psychological well-being after a hysterectomy are generally very good, which is likely due primarily to the reduction in previously existing symptoms [40–46]. Detailed studies on the extent to which organ loss affects well-being are rare. The literature on the subject is sparse overall, with most studies reporting no significant differences in sexual function between the two surgical methods. To our knowledge, there is currently just one study with a follow-up period of several years, which shows similar results as our study [47]. In this study, we show that, overall, there is no significant difference in the FSFI total score in the long-term follow-up, and thus TLH and LASH deliver equivalent results in terms of sexual function. This confirms the results of previous studies in a long-term follow-up. In TLH, there are no significant changes when comparing the baseline data with the follow-up data. In LASH, on the other hand, there is a significant deterioration in lubrication and in the total score over the follow-up period, even if this does not lead to a statistically significant difference in the total score when comparing LASH and TLH. Nevertheless, it should be noted that sexual function declined significantly more over time in the LASH group than in the TLH group. Whether this deterioration in the LASH group is solely due to the deterioration in lubrication, and whether this is directly related to the surgical method alone or whether it is a multifactorial event involving parameters that were not recorded in this study, cannot be conclusively clarified. It is interesting to note that lubrication is the only parameter in either group that shows a significant deterioration at follow-up, at least in the LASH group (4.96 vs. 3.62, p-value: 0.007). In the TLH group, the lubrication score also decreases, but does not show a significant change (4.32 vs. 3.90, p-value: 0.259). It is also interesting that the change in lubrication leads to a significant decrease of the total score in the LASH group (27.61 vs. 22.25, p-value: 0.028) but this detoriation in the LASH group compared to the TLH group does not result in a significant change in the total score (22.25 vs. 22.51, p-value: 0.261).

### Limitations

This study has several limitations that must be taken into account when interpreting the results. First, it is a questionnaire-based survey that is potentially influenced by recall bias, as the information on sexual function is based on subjective self-reports from the patients. In addition, participation was voluntary, which means that selection bias among participants cannot be ruled out. Furthermore, potential influencing factors such as hormonal status or psychological comorbidities were not adequately recorded. Finally, the sample size is limited, which restricts the significance of the results, particularly for any subgroup analyses. In addition, attrition may have occurred, as there are significantly fewer complete follow-up results for the LASH group (n = 29) than for the TLH group (n = 45).

## Conclusion

In summary, both surgical methods are safe techniques that ensure comparable postoperative sexual function, even in long-term follow-up. No statistically significant differences were found with regard to the overall outcome of the FSFI. However, LASH shows a decreasing trend in lubrication over time, which is also reflected in a deterioration in the overall score, but without this being expressed in a significant deterioration compared to the overall score of TLH.

## Data Availability

No datasets were generated or analysed during the current study.
